# The Pharmacokinetics of Saliva and Plasma N-Oxides Following a Single Administration of a Plant-Based Bioequivalent Inorganic Nitrate Oral Supplement in an Open-Label, Phase 1, Single-Arm Study

**DOI:** 10.3390/jcm14134581

**Published:** 2025-06-27

**Authors:** Macy E. Stahl, Emily E. Grammer, Jason D. Allen, Arthur Weltman

**Affiliations:** 1 Department of Kinesiology, University of Virginia, Charlottesville, VA 22903, USA; mes4hm@virginia.edu (M.E.S.); erk9nk@virginia.edu (E.E.G.); ja6af@virginia.edu (J.D.A.); 2 School of Medicine, University of Virginia, Charlottesville, VA 22903, USA

**Keywords:** nitric oxide, saliva, plasma, nitrate, nitrite, pharmacokinetics, elevated blood pressure, open-label, phase 1

## Abstract

**Background/Objective:** Hypertension and other modifiable risk factors for cardiovascular disease are characterized by a dysfunctional vascular endothelium and decreased nitric oxide (NO) bioavailability. The oral supplementation of inorganic nitrate (NO_3_^−^) has been shown to increase the salivary and plasma nitrite (NO_2_^−^), a precursor to NO, though there may be significant variation in the pharmacokinetics of this process between different supplements. The purpose of this open-label, phase 1, single-arm study was to investigate the pharmacokinetic profile of the plasma and salivary NO_3_^−^ and NO_2_^−^ concentrations following the administration of a single serving of a plant-based bioequivalent inorganic nitrate oral supplement (“Berkeley Life Nitric Oxide Foundation Capsules”, Chicago, IL, USA). **Methods:** Nine males and three females (age: 33 ± 15 years; BP: 129 ± 6 mmHg; BMI: 27.58 ± 4.27 kg/m^2^) participated in the protocol. Following the baseline collection of saliva and plasma samples, the participants consumed 314 mg (two capsules) of the supplement. Saliva and plasma samples were collected at 2 h, 4 h, 8 h, and 24 h post consumption. **Results:** The peak salivary NO_3_^−^ (13,326.12 ± 4926.60 µM), salivary NO_2_^−^ (1375.27 ± 679.28 µM), plasma NO_3_^−^ (498.37 ± 168.89 µM), and plasma NO_2_^−^ (231.66 ± 97.26 nM) were observed at 2 h post-supplementation (*p* < 0.01 vs. the baseline). The concentrations of the salivary and plasma NO_2_^−^ remained elevated at 8 h after administration (220% and 50% above the baseline, respectively), and the concentrations of the salivary and plasma NO_3_^−^ remained elevated at 24 h after administration (22% and 50% above the baseline, respectively). **Conclusions:** These data suggest that a single serving of “Berkeley Life Nitric Oxide Foundation Capsules” taken once to twice per day is a viable strategy to provide sustained salivary and plasma NO_3_^−^ and NO_2_^−^ availability over 24 h and therefore may provide a viable approach for long-term blood pressure maintenance.

## 1. Introduction

Elevated blood pressure (BP), defined as a resting blood pressure of >120/80 mmHg, is a leading attributable cause of death worldwide. In the United States, nearly half (48%) of adults have high blood pressure, yet only one in four (22.5%) of those have their BP under control [1]. Hypertension and other modifiable risk factors for cardiovascular disease (CVD), including diabetes and inflammation, are often marked by disruptions in the vascular endothelium and decreased nitric oxide (NO) bioavailability [2,3].

Recently, an alternative exogenous route to increase NO bioavailability has been explored via the oral supplementation of inorganic nitrate (NO_3_^−^) and its subsequent conversion to bioavailable nitrite (NO_2_^−^) [4]. Large clinical trials and several comprehensive reviews on the role of NO_3_^−^ in cardiovascular health have been published, showing blood-pressure-lowering effects in healthy and hypertensive individuals [5,6,7].

There is consensus on the principal pathways and primary mechanisms involved in the conversion of oral NO_3_^−^ to circulating NO_2_^−^ and NO [8]; however, there remains significant variation in the levels of NO metabolites obtained in the saliva and plasma that is dependent upon the type of oral inorganic NO_3_^−^ supplementation. The reduction of inorganic NO_3_^−^ to bioavailable NO is a two-cycle process: (1) Inorganic NO_3_^−^ is swallowed and absorbed into the circulation, where a proportion is then sequestered and concentrated in the salivary glands and slowly released into the oral cavity. At this stage, commensal bacteria reduce NO_3_^−^ to NO_2_^−^ in the oral cavity. (2) The newly formed salivary NO_2_^−^ is swallowed and absorbed into the circulation. The circulating NO_2_^−^ is then readily reduced to NO via a variety of pathways involving single-electron transfer reactions with protons (H+) and hemeproteins (i.e., hemoglobin, myoglobin) during deoxygenation [9].

A key step in this process is the reduction of NO_3_^−^ to NO_2_^−^ in the mouth, which is highly reliant on the bacterial species within the oral microbiome [10]. In fact, we (and others) have shown that the disruption of the oral microbiome using commercially available antibacterial mouthwash prevented increases in the plasma NO_2_^−^ following oral inorganic NO_3_^−^ supplementation and blunted reductions in the blood pressure compared to the control conditions [11,12,13]. While the salivary NO_2_^−^ is correlated with the plasma NO_2_^−^, the concentrations are 1000 times greater in the saliva; thus, salivary concentration does not accurately reflect the more directly bioavailable concentrations in the vascular compartment.

Previously named “Berkeley Life Nitric Oxide Foundation Capsules” (Chicago, IL, USA), a plant-based bioequivalent inorganic NO_3_^−^ dietary supplement was administered (two capsules containing 314 mg inorganic NO_3_^−^ taken once daily) for 12 weeks in a randomized, placebo-controlled, single-center, double-blinded study to 67 adults (40–67 years) with a BP > 120/80 mmHg who were on a stable hypertensive medication regimen (NCT03909789). The study showed a significant increase in the saliva and plasma NO_3_^−^ and NO_2_^−^ and a reduction in the systolic and diastolic blood pressure (12.5 ± 13.3 and 4.7 ± 10.3 mmHg, respectively, both *p* < 0.01) in the “active” capsule group [14]. However, this study only sampled the saliva and plasma NO_3_^−^ and NO_2_^−^ at 2 h post consumption; thus, the pharmacokinetics of this supplement beyond 2 h have not been elucidated. Understanding the pharmacokinetics of a single dose over a 24 h period will help determine optimal dosing strategies for achieving sustained elevations in the plasma and salivary NO_3_^−^ and NO_2_^−^.

The purpose of the present study was to examine the pharmacokinetic profile of plasma and salivary NO_3_^−^ and NO_2_^−^ concentrations following the administration of a single serving of 314 mg (two capsules) of a plant-based bioequivalent inorganic nitrate oral supplement (“Berkeley Life Nitric Oxide Foundation Capsules”, Chicago, IL, USA).

## 2. Materials and Methods

### 2.1. Study Design

**Figure 1:**
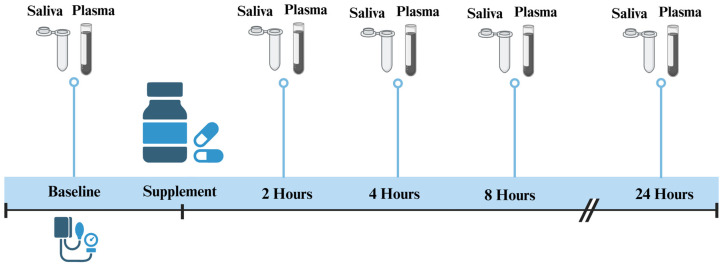
Study design. Figure created in Biorender.

This was an open-label, phase 1, single-arm study that included 12 adults with elevated blood pressure (resting BP > 120/80 mmHg) but who were otherwise apparently healthy. Following the subjects’ provision of written informed consent, they underwent a health history survey, the collection of saliva and plasma samples for the analysis of NO_3_^−^ and NO_2_^−^ concentrations, and baseline resting blood pressure assessments. Blood and saliva measurements were taken at baseline (0) and 2, 4, 8, and 24 h following capsule administration (as outlined in Figure 1).

### 2.2. Preparation and Content of Capsules

The Berkeley Life Nitric Oxide Foundation Capsules were provided by Berkeley Life (Chicago, IL, USA), with the following active ingredients: Vit C, 290 mg; thiamin (from thiamin mononitrate), 90 mg; Vit B12 (as methylcobalamin), 200 mcg; magnesium (from magnesium citrate) 75 mg; potassium (from potassium nitrate), 189 mg; and a Proprietary Blend (potassium nitrate, beetroot extract [25% betaine nitrate], organic fermented beetroot power), 500 mg. The formula was encapsulated in a capsule made of hydroxypropyl methycellulose, Nu-Mag (rice extract blend), Nu-Rice (Rice Bran Extract), and Nu-Flow (rice hulls). The contents of the capsules were verified by mass spectroscopy. The current Berkeley Life Nitric Oxide Foundation Capsules used in this study were equivalent to those used previously for 12 weeks with no reported adverse effects [14].

### 2.3. Participants

The study was completed over a three-month period (February–April 2024). Participants were recruited from the University of Virginia and the surrounding Charlottesville, Virginia, area and were primarily identified through snowball sampling. All the procedures were approved by the Institutional Review Board at the University of Virginia (HSR#230481), and the study was conducted in accordance with the Declaration of Helsinki. The study was registered on ClinicalTrials.gov (NCT06777108). All the participants were given a written version of the protocol to read and were provided with an opportunity to ask questions prior to providing written informed consent.

The inclusion criteria were individuals (a) over 18 years old, (b) with a BP > 120/80, (c) who were able to provide written informed consent and (d) had an absence of any significant cardiac or other medical history, and (e) had no medication changes in the preceding six months. The exclusion criteria were (a) a history of oral cancer, (b) tobacco smokers, (c) individuals taking nitroglycerine (or inorganic NO_3_^−^), PDE-5 inhibitors (ex: Cialis, Viagra), and xanthine oxidase inhibitors (ex: Allopurinol), and (d) individuals who had used oral antibiotics or an antibacterial/antimicrobial mouthwash within the previous four weeks. Thirteen individuals were screened for the protocol, though one did not meet the eligibility criteria due to a recent medication change. Therefore, 12 individuals received the intervention and completed the following protocol over a 24 h period.

The participants were asked to refrain from consuming foods rich in NO_3_^−^ (e.g., beets, leafy greens, etc.) for at least 48 h and to refrain from vigorous exercise and drinking caffeine and alcohol for at least 12 h prior to the visit. They arrived at the University of Virginia Student Health and Wellness Building at ~9 am in a fasted state and without brushing their teeth or using mouthwash that morning. Upon arrival, the participants completed a health history questionnaire prior to the baseline BP reading and oral and blood sample collections. The participants then consumed two Berkeley Life Foundation Capsules (total of 314 mg inorganic NO_3_^−^) with a small amount of water. Saliva and blood samples were collected in an identical fashion at baseline (0) and at 2, 4, 8, and 24 h following capsule administration.

### 2.4. Saliva and Plasma Sample Collection

At each timepoint, the participants underwent an unstimulated saliva sample collection into a sterile tube using a passive drool technique in which individuals aimed to minimize the churning of saliva or spitting. The samples were immediately placed in a −80 deg. Celsius freezer for subsequent analysis. Blood draws were obtained at the same timepoints from an antecubital vein and placed into two, 4 mL lithium heparin tubes (Vacuette tubes, REF 454029)(Greiner Bio-One, Monroe, NC, USA). The blood samples were immediately centrifuged for five minutes, and the plasma was removed and aliquoted into two, 2 mL Eppendorf tubes (1.7 mL Axygen, cat# MCT-175-C) (Corning, Glendale, AZ, USA) and placed in a −80 deg. Celsius freezer for subsequent analysis.

For comparison, at each timepoint, a Berkeley Life Nitric Oxide Saliva Test strip was placed on the participant’s tongue for 5 s and then removed and folded for 5 s, as per the manufacturer’s instructions. The test strip estimations of the salivary NO_2_^−^ concentrations were immediately calculated using the calibration values of depleted, low, threshold, target, and high, which translated to 21, 108, 217, 434, and 869 μM NO_2_^−^, respectively.

### 2.5. NO_3_^−^ and NO_2_^−^ Analysis

At a later date, the plasma and saliva samples were batch-thawed and assessed for concentrations of NO_3_^−^ and NO_2_^−^ via ozone-based chemiluminescence using a Sievers Nitric Oxide Analyzer (NOA) model 280i (GE Analytical Instruments, Boulder, CO, USA). Prior to each analysis, a standard curve prepared via a serial dilution was run to calibrate the NOA to the NO_3_^−^ and NO_2_^−^ measurements. All the concentrations of NO_3_^−^ and NO_2_^−^ in the saliva and plasma samples fell within the bounds of the standard curve, and a standard solution was injected at the beginning and end of each run to ensure the consistency of the sampling.

Following the dilution of the saliva with deionized water in a 1:100 ratio (saliva:deionized water), the NO_2_^−^ concentrations in the saliva and plasma were measured as per the manufacturer’s instructions in the presence of glacial acetic acid and potassium iodide.

The plasma samples were analyzed for their NO_3_^−^ concentrations following deproteinization using cold ethanol in a 1:3 dilution (plasma:ethanol). The samples were then incubated on ice for 30 min before being centrifuged at 14,000× *g* for 10 min. The saliva samples were similarly diluted with deionized water in a 1:100 ratio. The resulting plasma supernatant and diluted saliva samples were analyzed as per the manufacturer’s instructions in the presence of vanadium chloride dissolved in 1 N HCl at 95 °C.

### 2.6. Statistical Analysis

Serial measures of the plasma and salivary NO_3_^−^ and NO_2_^−^ were analyzed using a one-way analysis of variance (ANOVA) with repeated measures and a correction for multiple comparisons. For pharmacokinetic modeling, the mean timepoint data were imported into Matlab (version R2023a, Mathworks, Natick, MA, USA) and interpolated at every half-hour mark using cubic Hermite spline interpolation (Matlab interp1 function with the pchip option).

Paired *t*-tests and Pearson product moment correlations were used to compare the salivary NO_2_^−^ values obtained using the Berkeley Life Nitric Oxide Saliva Test strips and those measured using ozone-based chemiluminescence. The data is reported as the means ± the standard deviations unless otherwise noted, and *p* < 0.05 was required for statistical significance.

## 3. Results

### 3.1. Participant Characteristics

The study population consisted of nine males and three females with ages ranging from 21 to 60 years (33 ± 15 years old) and with BMIs of 21.5–35 (27.58 ± 4.27 kg/m^2^). Their resting systolic blood pressures (SBPs) ranged between 122 and 142 mmHg (129 ± 6 mmHg) and their diastolic blood pressures (DBPs) ranged between 82 and 88 mmHg (83 ± 2 mmHg). The capsule dosages and administration were well-tolerated with no adverse effects reported. Blood could not be collected at 24 h for one participant, resulting in the collection of 11 samples at this timepoint. Otherwise, saliva and plasma samples were collected successfully at all the timepoints from all 12 participants.

### 3.2. Saliva and Plasma NO_3_^−^

**Figure 2:**
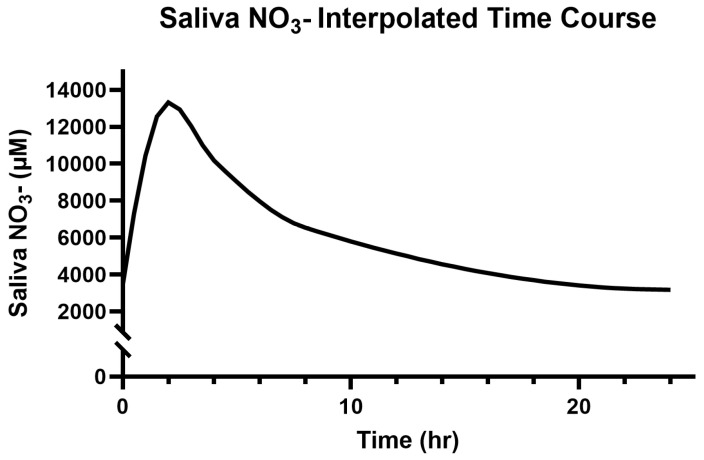
Modeled time course for saliva NO_3_^−^ over 24 h.

**Figure 3:**
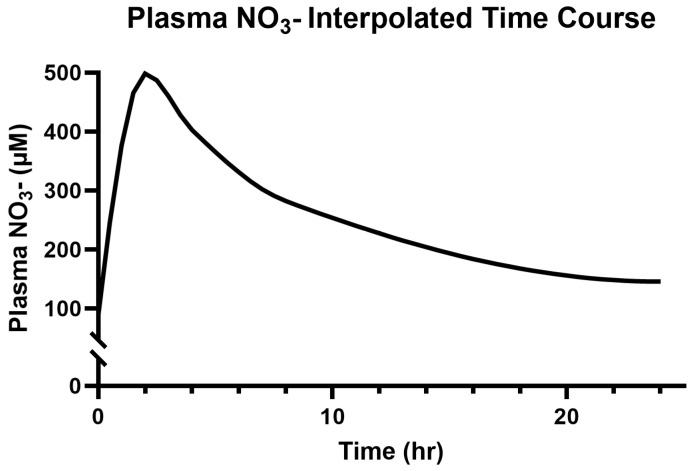
Modeled time course for plasma NO_3_^−^ over 24 h.

The participants’ baseline salivary and plasma NO_3_^−^ concentrations were 3472.70 ± 3143.22 µM and 89.01 ± 32.05 µM, respectively. The modeled and interpolated time course responses to 314 mg (two capsules) of Berkeley Life Pro over the 24 h period can be seen in Figure 2 and Figure 3 below.

The peak salivary (13,326.12 ± 4926.60 µM) NO_3_^−^ concentrations were recorded at 2 h (*p* < 0.01 vs. the baseline) and remained elevated above the baseline for 8 h (*p* < 0.05) after capsule ingestion. The estimated values were elevated above the baseline by 50% and 25% at 11.5 h and 15 h, respectively.

The plasma NO_3_^−^ concentrations peaked at 2 h post consumption (498.37 ± 168.89 μM, *p* < 0.001) and remained elevated (50%) above the baseline at the 24 h timepoint (*p* < 0.05).

### 3.3. Saliva and Plasma NO_2_^−^

**Figure 4:**
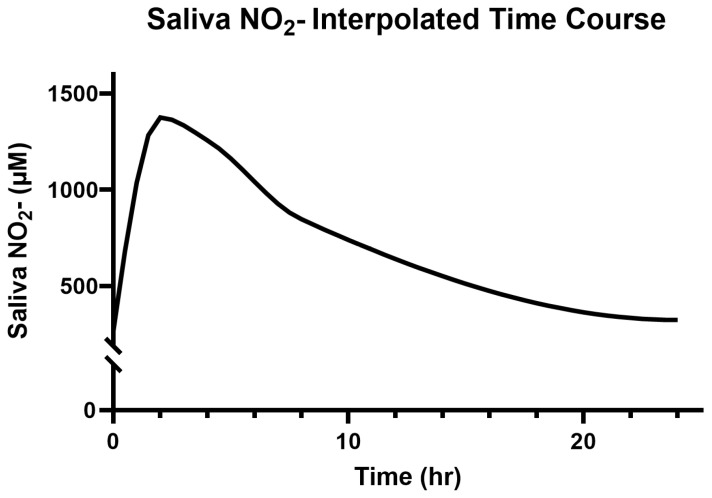
Modeled time course for saliva NO_2_^−^ over 24 h.

**Figure 5:**
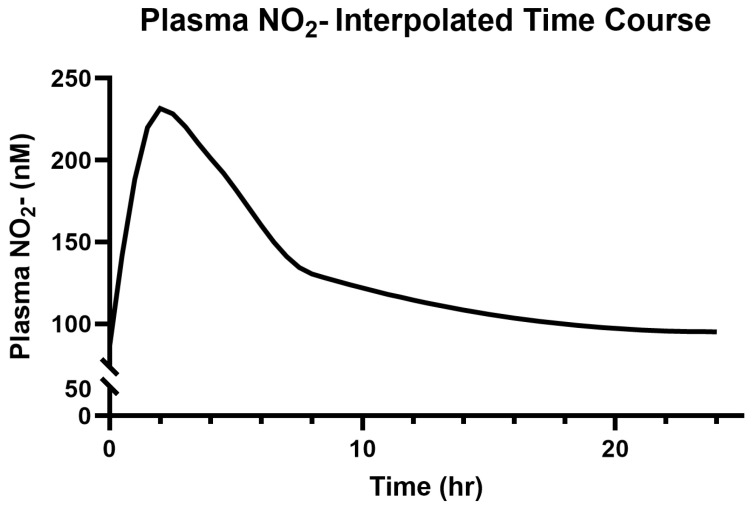
Modeled time course for plasma NO_2_^−^ over 24 h.

Participants’ baseline salivary and plasma NO_2_^−^ concentrations were 264.4 ± 149.9 µM and 86.5 ± 15.6 nM, respectively. The modeled and interpolated time course responses to 314 mg (two capsules) of Berkeley Life Pro over the 24 h period can be seen in Figure 4 and Figure 5.

The peak salivary NO_2_^−^ (1375.27 ± 679. 28 µM) occurred at the 2 h timepoint (*p* < 0.01 vs. the baseline) and remained elevated for over 8 h (*p* < 0.01) after capsule ingestion. The estimated values were elevated above the baseline by 50% and 25% at 18.5 h and 22 h, respectively (Figure 4).

The peak plasma NO_2_^−^ (231.66 ± 97.26 nM) was recorded at 2 h (*p* < 0.01 vs. the baseline) and remained elevated for over 8 h (*p* < 0.01) after capsule ingestion. The estimated values were elevated above the baseline by 50% and 25% at 8 h and 14 h, respectively (Figure 5).

### 3.4. Saliva Test Strips and Chemiluminescence

**Figure 6:**
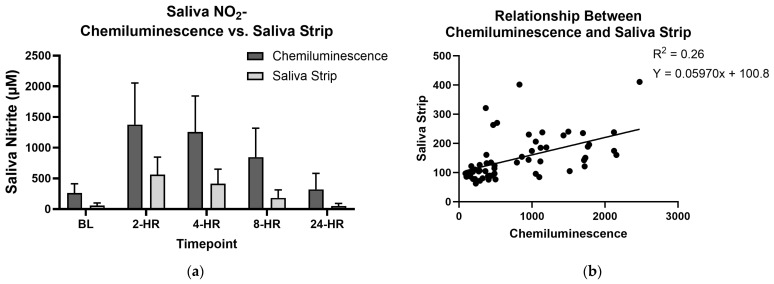
Saliva test strips versus chemiluminescence: (**a**) comparison between saliva NO_3_^−^ concentrations obtained using Berkeley Life saliva test strips and ozone-based chemiluminescence using Sievers NOA model 280i; (**b**) correlation between results obtained using Berkeley Life saliva test strips and ozone-based chemiluminescence.

The salivary NO_2_^−^ concentrations calculated from the Berkeley Life Test Nitric Oxide saliva test strips were lower than those observed via ozone-based chemiluminescence, as can be seen in Figure 6. However, the test strip values were correlated with those measured via ozone-based chemiluminescence (Figure 6b, *p* < 0.01), indicating that both methods differentiated between higher and lower salivary nitrite levels.

## 4. Discussion

Vascular NO bioavailability is essential for cardiovascular health, and a reduction in the ability of the vascular endothelium to produce NO is an early event in the process of atherosclerotic lesion formation and is associated with cardiovascular risk factors [15,16], diabetes [3,17], and established cardiovascular disease [18]. The administration of oral NO_3_^−^ and subsequent increases in the level of N-oxides in the saliva and blood have demonstrated a reduction in blood pressure in individuals with hypertension [5,6,14,19,20]. Despite a consensus on the primary pathways and mechanisms involved in the conversion of oral NO_3_^−^ to circulating NO_2_^−^ and NO, there is a dearth of reports on the pharmacokinetics of different formulations or modes of administration. The short half-life of NO makes it difficult to measure directly in vivo, but its expression has previously been shown to be directly proportional to the plasma NO_2_^−^ levels [21,22], suggesting that this marker may be a measurable reflection of the vascular NO bioavailability.

The current study showed that 314 mg (two capsules) of Berkeley Life Foundation Capsules increased the plasma NO_2_^−^ rapidly (within 2 h), and the values stayed 50% higher than the baseline for at least 8 h and were estimated to be elevated by 25% above the baseline at 14 h post consumption. This suggests that a dose of 314 mg (two capsules) taken every 12 h (BID) is likely to maintain an elevated NO_2_^−^ concentration in the vascular compartment.

Notably, while the levels of all N-oxides measured in this study increased and declined following oral NO_3_^−^ administration, they did so with different pharmacokinetic profiles, indicating differences in their digestion/production and subsequent conversion/excretion. For example, when inorganic NO_3_^−^ is swallowed and circulating in the plasma, only about 20% is sequestered in the salivary glands, but it becomes greatly concentrated [23]. This was shown by the salivary NO_3_^−^ values being almost 40 times higher than those in the plasma at baseline (un-supplemented) and 26 times higher 2 h post-supplementation. Bacteria in the oral cavity then convert NO_3_^−^ to NO_2_^−^, which can be dependent upon the species and their abundance in an individual’s oral microbiome [24]. In our sample, the rate of conversion appeared to be approximately 10%. Most noteworthy is that the concentration of the plasma NO_2_^−^ (nM) was at least 1 × 10^−3^ lower than that for the salivary NO_2_^−^ and plasma and saliva NO_3_^−^ (all in µM). This indicates that although all the values may rise and fall in a similar manner, they cannot be used interchangeably to indicate the vascular NO bioavailability.

The majority of the previous studies in this area have utilized beetroot juice as a source of inorganic NO_3_^−^ [25,26]. Commercial beetroot juice beverages typically have limited ingredients (beetroot and lemon juice) and contain 300–400 mg of inorganic NO_3_^−^. The capsules used in the current study, however, provide a combination of beetroot and potassium NO_3_^−^ at a slightly lower dose (314 mg), along with a variety of added vitamins (Vit C, thiamin, Vit B12, magnesium, and potassium). It is unclear from the present study how these additional ingredients contribute to the differences in pharmacokinetics, and a protocol directly comparing the two supplements is necessary to determine these differences, though they appear to result in similar NO_3_^−^ and NO_2_^−^ peak times around 2–3 h post-supplementation [27,28]. Berkeley Life Foundation Capsules may provide more clinical attractiveness, as they are less expensive and are more tolerable in terms of their taste and ease of consumption compared to beetroot juice [29,30]. Additionally, 10–14% of individuals experience the discoloration of their urine following the consumption of beetroot juice, and others have reported dark or red stools [31,32]. These effects have not been reported for Berkeley Life Foundation Capsules, suggesting that they may be a suitable alternative for chronic consumption.

It appears that the most consistently observed clinical outcome following increased oral inorganic NO_3_^−^ intake is a reduction in blood pressure in subjects with and without hypertension [20,33,34]. The clinical significance of BP reduction is brought into perspective when we consider that a 1 mmHg increase in SBP is estimated to increase the cerebrovascular incident mortality by 2% and a 1 mmHg increase in DBP may increase the stroke mortality by 3% [35,36]. Previous data from a study using Berkeley Life Pro Capsules (314 mg inorganic NO_3_^−^; two capsules taken once daily) for 12 weeks showed a 12.5 ± 13.3 mmHg reduction in the SBP and a 4.7 ± 10.3 mmHg reduction in the DBP approximately 2 h after consumption [14].

Though the current sample showed an absence of major cardiovascular risk factors aside from hypertension, chronic supplementation may be a valuable way to control blood pressure via the elevation of N-oxides in the plasma, which can be readily converted to NO. Given that the World Health Organization estimates that hypertension affects 33% of adults aged 30–79 worldwide and a high systolic BP is responsible for approximately one in five deaths, harnessing the blood-pressure-lowering effects of inorganic NO_3_^−^ supplementation may be a relatively inexpensive and simple approach to reducing risk progression [33]. These results may also have clinical utility in other populations that have reduced endogenous NO bioavailability, including patients with cardiovascular disease and postmenopausal females [37,38,39,40,41,42,43], and future studies should evaluate the effectiveness of Berkeley Life Foundation Capsules in these populations.

## 5. Limitations

This study is not without limitations. A key limitation is the study sample size of 12 participants. As this was a preliminary study examining the pharmacokinetics of an oral nitrate supplement, we did not base the sample size on previous studies; thus, the results should be interpreted with caution. Additionally, the present study was conducted in individuals with elevated blood pressure who were otherwise apparently healthy. As such, these data should only be generalized to other clinical populations with caution, and future studies exploring the pharmacokinetic profile of Berkeley Life Capsules in other cohorts are necessary. Another limitation of the study is that we did not compare dosages (e.g., one, two, three, etc., capsules); thus, we are unable to report whether the capsules have differing conversion rates at different doses.

## 6. Conclusions

Vascular NO bioavailability is essential for cardiovascular health, and oral NO_3_^−^ supplementation is a viable method to increase NO species. The data from this study shows that the ingestion of two Berkeley Life Pro Capsules containing 314 mg inorganic NO_3_^−^ results in a significant elevation in the salivary and plasma NO_2_^−^ and NO_3_^−^ for 8 h, suggesting a sustained pool of bioavailable NO. In combination with the previous literature displaying an improvement in blood pressure following chronic supplementation with Berkeley Life Foundation Capsules [14], it is suggested that taking two capsules (314 mg of NO_3_^−^) once to twice daily is an acceptable strategy to provide sustained salivary and plasma NO_2_^−^ and NO_3_^−^ availability over 24 h and as such may be a viable approach for long-term blood pressure maintenance.

Furthermore, the saliva nitrite strips, while not quantitatively accurate compared to lab testing, provide a convenient method for indicating a gradient of salivary NO_2_^−^ levels.

## Data Availability

The original contributions presented in this study are included in the article. Further inquiries can be directed to the corresponding author(s).

## Abbreviations

The following abbreviations are used in this manuscript:

NO	Nitric Oxide
NO_3_^−^	Nitrate
NO_2_^−^	Nitrite
BP	Blood Pressure
SBP	Systolic Blood Pressure
DBP	Diastolic Blood Pressure
CVD	Cardiovascular Disease
